# Highly Pathogenic Avian Influenza Virus Infection in Feral Raccoons, Japan

**DOI:** 10.3201/eid1704.101604

**Published:** 2011-04

**Authors:** Taisuke Horimoto, Ken Maeda, Shin Murakami, Maki Kiso, Kiyoko Iwatsuki-Horimoto, Mariko Sashika, Toshihiro Ito, Kazuo Suzuki, Mayumi Yokoyama, Yoshihiro Kawaoka

**Affiliations:** Author affiliations: University of Tokyo, Tokyo, Japan (T. Horimoto, S. Murakami, M. Kiso, K. Iwatsuki-Horimoto, Y. Kawaoka);; Yamaguchi University, Yamaguchi, Japan (K. Maeda);; Obihiro University of Agriculture and Veterinary Medicine, Hokkaido, Japan (M. Sashika);; Tottori University, Tottori, Japan (T. Ito);; Hikiiwa Park Center, Wakayama, Japan (K. Suzuki);; Wildlife Management Research Center, Hyogo, Japan (M. Yokoyama)

**Keywords:** Viruses, influenza virus, H5N1, avian influenza, raccoon, Japan, dispatch

## Abstract

Although raccoons (*Procyon lotor*) are susceptible to influenza viruses, highly pathogenic avian influenza virus (H5N1) infection in these animals has not been reported. We performed a serosurvey of apparently healthy feral raccoons in Japan and found specific antibodies to subtype H5N1 viruses. Feral raccoons may pose a risk to farms and public health.

Although all known subtypes of influenza A virus are maintained in waterfowl, these viruses have also been isolated from various avian and mammalian species. In particular, numerous reports have been made of highly pathogenic avian influenza viruses (H5N1) infecting mammals, causing lethal infections in some species (*1**,**2*). Wild mammals could transmit these viruses among other wild and domestic animals, for example, on poultry or pig farms, posing a risk for virus spread and the emergence of mutant viruses. Such viruses could have pandemic potential if they were able to infect humans, thus giving rise to a serious public health concern. Therefore, the continuous monitoring of the exposure of wild mammals to avian influenza viruses, particularly H5N1 viruses, is essential.

Raccoons (*Procyon lotor*), which belong to the *Carnivora*, are native to North America. Since the 1970s, a large number of raccoons have been imported as pets into Japan. The release and escape of these animals have resulted in a feral population widely distributed throughout Japan, which continues to increase despite an official eradication program. Recent reports, including serologic surveys and experimental infections, indicate that raccoons can be symptomatically or asymptomatically infected with low pathogenic influenza viruses, such as avian influenza subtype H4N8 or human influenza subtype H3N2 viruses, which they shed for several days, resulting in virus transmission to other raccoons by aerosol (*3**–**5*). Such findings present the possibility that wild raccoons could play a role in the transmission of subtype H5N1 viruses in a natural setting. We conducted a serologic survey for subtype H5N1 virus infection in feral raccoons in Japan.

## The Study

**Figure 1:**
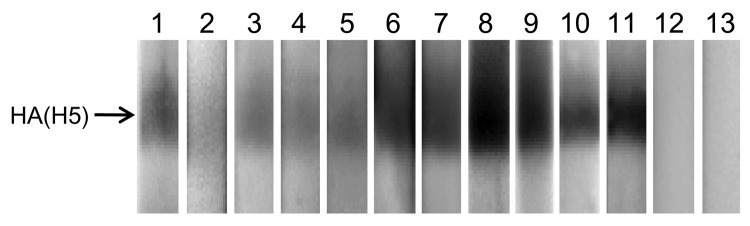
Western blot analysis of virus neutralization (VN)–positive raccoon serum specimens. A/whooper swan/Mongolia/4/05 (H5N1) virus (clade 2.2) was purified through a 25% sucrose cushion and used as an antigen under nonreducing conditions in the Western blot assay. After blocking with 5% skim milk, each raccoon serum specimen (1:100 dilution) was incubated for 1 h and then reacted with horseradish peroxidase (HRP)–labeled protein A/G (Pierce Chemical Co., Rockford, IL, USA) and subjected to chemiluminescence detection (ECL Plus, GE Healthcare UK, Ltd, Chalfont St. Giles, UK). Serum from a mouse infected with A/whooper swan/Mongolia/4/05 was used as a marker. The negative control reaction when only HRP-protein A/G is used is also shown. HA, hemagglutinin; Ab, antibody. Lane 1, anti-H5N1 polyclonal Ab; lanes 2–11, VN-positive serum (2, A-1; 3, A-2; 4, A-3; 5, A-4; 6, A-5; 7, A-6; 8, B-1; 9, B-2; 10, C-1; 11, C-2); lane 12, VN-negative serum; lane 13, negative control.

Raccoons are considered an invasive alien species in Japan. Recently, the growing population of feral raccoons has resulted in significant agricultural damage and prompted the initiation of eradication programs in several areas. We used a total of 1,088 serum samples collected from animals captured under this official eradication program over 3 periods in the western region of Japan and 1 period in eastern Japan during 2005–2009 for a serologic survey of avian influenza virus (H5N1) infection (Table 1). To detect antibodies specific to the H5 hemagglutinin (HA) in the serum samples, we performed a virus neutralization (VN) test (*6*) with 2 subtype H5N1 viruses, A/Indonesia/3006/2005 (clade 2.1.3) and A/whooper swan/Mongolia/4/2005 (clade 2.2). As an initial screening step, we used the serum specimens (1:5 dilution) after receptor-destroying enzyme treatment of the serum to remove nonspecific inhibitors. The VN antibody-positive serum samples were then further tested for their reactivity by using a panel of influenza viruses of multiple subtypes (Table 2) as well as Western blot analysis (Figure 1). In these assays, we found a total of 10 serum specimens that were positive for VN antibody to subtype H5N1 viruses, representing 0.9% positivity. The A-1 to A-6 serum specimens, which were collected from animals captured within a 10 km^2^ area, strongly reacted to A/whooper swan/Mongolia/4/2005 (clade 2.2) and more weakly to other clades of subtype H5N1, H5N2, and H5N3 viruses. These serum specimens did not react to viruses of other HA subtypes, including H1, H3, H7, and H9. Of note, the A-2, A-3, and A-4 animals were from the same litter captured at a lair, which suggests that the detected VN antibodies in these samples might be maternal antibodies from their uncaptured mother, who may have been infected with a subtype H5N1 virus. It is possible that 2 viruses of clade 2.2, which had slightly different antigenicities, may have infected raccoons in this area, as indicated by the different patterns of cross-reactive VN titers to subtype H5N1 clade 1 and H5N3 viruses. One group consisted of A-1 to A-4 and the other of A-5 and A-6. The B-1 and B-2 samples from animals captured at a 25-km distance strongly reacted to both subtype H5N1 clades 2.2 and 2.5 viruses. Given that the subtype H5N1 clade 2.5 virus has not circulated since 2004 and that the clade 2.2 virus was more highly reactive than the clade 2.5 virus, these raccoons were likely infected with clade 2.2 viruses, as supported by timing with poultry outbreaks. By contrast, the C-1 and C-2 samples from raccoons captured in eastern Japan reacted strongly to A/whooper swan/Akita/1/2008 (clade 2.3.2), unlike the samples from western Japan, indicating that the C-1 and C-2 animals were infected with a virus of this clade. Together, these data suggest that feral raccoons in Japan have been infected with subtype H5N1 viruses of different clades.

**Table 1:** Summary of serologic test results showing avian influenza (H5N1) antibody–positive samples from feral raccoons, Japan*

Region/period	No. positive/no. samples (%)	ID no., positive samples	Date raccoon captured	Animal body mass, kg	Animal sex
Western Japan					
2005 May–2006 Dec	6/221 (2.7)				
		A-1	2006 Apr 30	5.6	F
		A-2	2006 May 2	0.3	M
		A-3	2006 May 2	0.3	M
		A-4	2006 May 2	0.3	M
		A-5	2006 May 17	10.9	M
		A-6	2006 May 25	9.1	M
2007 Jun–2008 May	2/84 (2.4)				
		B-1	2007 Jun 28	5.3	M
		B-2	2008 Jan 20	7.2	M
2009 Apr–2009 Sep	0/110				
Eastern Japan					
2007 May–2008 Oct	2/683 (0.3)				
		C-1	2008 May 8	4.0	F
		C-2	2008 Jul 1	7.2	M
Total	10/1,088 (0.9)				

*ID, identification.

**Table 2:** Cross-reactivity of avian influenza (H5N1) virus neutralizing antibody–positive samples from feral raccoons, Japan*

ID no., positive samples	Virus antigen subtype			
	H1N1, H3N2, H7N6, H7N7, H9N2	H5N1 clade	H5N2	H5N3

1	2.1.3	2.2a	2.2b	2.3.2	2.3.4	2.5
A-1	<8	<8	8	32	8	<8	<8	16	<8	<8
A-2, 3, 4	<8	<8	64	256	64	<8	<8	128	<8	<8
A-5	<8	32	64	512	64	8	8	256	<8	64
A-6	<8	32	64	1,024	128	8	8	128	8	16
B-1	<8	32	32	256	128	8	16	256	8	64
B-2	<8	8	16	256	64	<8	8	256	8	64
C-1	<8	8	16	256	16	1,024	<8	64	<8	<8
C-2	<8	<8	16	512	16	256	<8	256	<8	<8

*We used A/PR8/34 (H1N1), A/Aichi/2/68 (H3N2), A/quail/Aichi/1/09 (H7N6), A/seal/MA/1/80 (H7N7), A/Hong Kong /1073/99 (H9N2), A/Vietnam/1194/04 (H5N1; clade 1), A/Indonesia/3006/05 (H5N1; clade 2.1.3), A/whooper swan/Mongolia/4/05 (H5N1; clade 2.2a), A/chicken/Miyazaki/K11/07 (H5N1; clade 2.2b), A/whooper swan/Akita/1/08 (H5N2; clade 2.3.2), A/Hanoi/30850/05 (H5N1; clade 2.3.4), A/chicken/Yamaguchi/8/04 (H5N1; clade 2.5), A/chicken/Ibaraki/1/05 (H5N2), and A/whooper swan/Shimane/499/83 (H5N3) as antigens for the virus neutralization test. Representative data from 3 independent experiments are shown.

**Figure 2:**
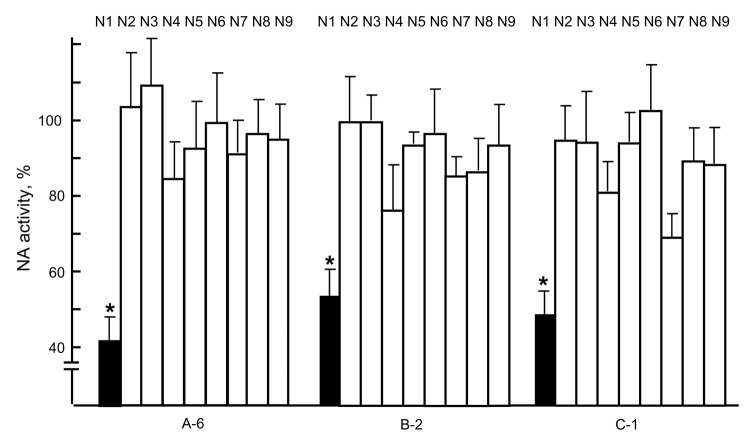
Neuraminidase (NA) inhibition by virus neutralization (VN)–positive raccoon serum samples. Each of the N1 to N9 viruses, consisting of A/whooper swan/Mongolia/4/05 (H5N1), A/Hong Kong/1073/99 (H9N2), A/whooper swan/Shimane/499/83 (H5N3), A/turkey/Ontario/6188/68 (H8N4), A/duck/Alberta/60/76 (H12N5), A/duck/England/56 (H11N6), A/seal/Massachusetts/1/80 (H7N7), A/duck/Ukraine/1/63 (H3N8), and A/duck/Memphis/546/74 (H11N9), was incubated with a VN-positive serum sample (A-6, B-2, or C-1) for 1 h at 37°C, and viral NA activity was then measured (*6*). Data are shown as percentage of activities compared with incubation with VN-negative serum samples (100%). Three independent tests were performed, and significant reduction of NA activity (p<0.05, *t* test with 2-tailed analysis) was observed only for N1 virus (*). HA, hemagglutinin. Error bars indicate SDs of 3 independent tests.

To assess the presence of anti-neuraminidase (NA) antibodies in the serum samples, we used an NA-inhibition (NI) assay for the VN-positive samples and found marked inhibition of the NA activity of the N1 subtype (Figure 2). We also performed the standard NI assay using another N1 virus, A/swine/Iowa/15/30 (H1N1), to avoid nonspecific NA inhibition by H5 antibodies, for 2 VN-positive serum specimens (A-6 and C-2) and found that A-6 and C-2 had positive NI titers of 20 and 80, respectively. These data demonstrate that VN-positive raccoon serum specimens contain anti-H5N1 antibodies, indicating that raccoons have been infected with subtype H5N1 viruses.

## Conclusions

Japan has experienced 3 outbreaks of highly pathogenic subtype H5N1 viruses. In the first in early 2004, clade 2.5 subtype H5N1 viruses were detected in poultry farms in western Japan. The second, in early 2007, involved the isolation of clade 2.2 subtype H5N1 viruses from poultry in western Japan. The third occurred in mid-2008, when clade 2.3.2 viruses were isolated from diseased swans in the lakes in the northern area of eastern Japan. All of these outbreaks were contained by prompt culling of birds. Since 2008, subtype H5N1 viruses have not been reported in any poultry or wild migratory birds under the government surveillance program. Our data indicate that raccoons in western Japan were likely infected with the clade 2.2 viruses, whereas those in eastern Japan were infected with the clade 2.3.2 virus. Notably, some antibody-positive raccoons in western Japan were captured 6 months before the poultry outbreak with clade 2.2 virus, suggesting that a clade 2.2 subtype H5N1 virus had invaded Japan by 2006.

We cannot determine by seropositive text results the exact date when the viruses infected the raccoons, because the duration of naturally acquired antibody to subtype H5N1 virus in this species is unknown. Recent data indicate that this animal maintains a detectable serum antibody response for at least 9 months after natural exposure to influenza viruses of other HA subtypes such as H1, H3, and H4 (*7*). In humans, a detectable antibody response to seasonal viruses can last >5 years (*8*) and in swine antibodies to the virus have been detected 28 months postinfection (*9*).

Because wild raccoons are omnivores and highly opportunistic at exploiting foods they prefer, whenever available they could eat diseased or dead migratory birds from areas where subtype H5N1 viruses are enzootic. They also sometimes attack poultry farms for food, creating the potential to transmit virus to domestic poultry. In addition, the increasing likelihood for contact between wild raccoons and humans elevates the possibility of human infection with these viruses, posing risks to public health and increasing the possibility of the emergence of mammalian-adapted mutant viruses with pandemic potential. Further investigation and surveillance of influenza virus infections in peridomestic animal species are needed to better understand influenza ecology.
